# Transition from disease to survival: accounts of adolescents who have experienced cancer

**DOI:** 10.1590/1518-8345.6302.3846

**Published:** 2022-11-28

**Authors:** Carolliny Rossi de Faria Ichikawa, Regina Szylit, Mariana Lucas da Rocha Cunha, Lisabelle Mariano Rossato, Elaine Cristina Rodrigues Gesteira

**Affiliations:** 1 Universidade de São Paulo, Escola de Enfermagem, Departamento de Enfermagem Materno-Infantil e Psiquiátrica, São Paulo, SP, Brazil; 2 Scholarship holder at the Conselho Nacional de Desenvolvimento Científico e Tecnológico (CNPq), Brazil; 3 Faculdade Israelita de Ciências da Saúde Albert Einstein, Hospital Israelita Albert Einstein, São Paulo, SP, Brazil; 4 Universidade Federal de São João Del Rei, Departamento de Enfermagem, Divinópolis, MG, Brazil

**Keywords:** Adolescent, Cancer Survivors, Neoplasms, Survivorship, Pediatric Nursing, Oncology Nursing

## Abstract

**Objective:**

to understand the transition from disease to survival of adolescents who had experienced cancer.

**Method:**

qualitative study, developed with the theoretical framework of symbolic interactionism, conducted with 14 adolescent cancer survivors treated at an outpatient clinic after cancer therapy, in the city of São Paulo. Individual in-depth interviews were performed and recorded, and the data were analyzed and interpreted using the methodological framework of the thematic analysis.

**Results:**

four themes were identified: going back to school, being able to live like other adolescents, living in the present moment, and seeking a purpose in life.

**Conclusion:**

the transition from disease to cancer survival was full of insecurities, difficulties, and challenges. After the disease, survivors acquire new values and new priorities in life, a reconstruction of the self. They also feel thankful to God and the people who were part of their treatment journey.

## Introduction

Until recently, cancer was considered a fatal disease. Currently, it has become a disease with high chances of survival^1^ ^), (^ ^2^. Childhood cancer treatment has improved significantly over the past 50 years, and therefore, the survival rate has increased steadily. Children and adolescents aged 0 to 19 years diagnosed with cancer have presented an increase of over 85% in the overall survival rate^2^ ^), (^ ^3^.

The concept of survivor was first described by a cancer survivor physician, Fitzhugh Mullan. In his article “Seasons of survival”, he defined survivor with a general idea that applies to everyone diagnosed with cancer, regardless of the course of the disease. For him, survival begins at the moment of diagnosis, because at that moment patients are forced to face their own mortality and begin to make the necessary adjustments for their future^4^. Mullan described three phases of survivorship: acute survivorship, the period when a person is diagnosed and treated; extended survivorship, the period after the end of treatment, when the patient handles the physical and psychological consequences of treatment; and permanent survivorship, the period in which remission is unlikely to happen^4^.

Survivor, in the biomedical concept, refers to an individual who has had a life-threatening disease and remains free of such disease for at least five years after finishing treatment^2^ ^), (^ ^3^. This is the concept used in this study.

With an increasing number of cancer survivors and high demand for health needs, the National Cancer Institute (NCI) created in 1996 the Office of Cancer Survivorship (OCS)^5^, which, in partnership with the National Coalition for Cancer Survivorship (NCCS), developed new guidelines for the care and research with cancer survivors. Through the document “Lost in transition”, the organizations described cancer survival as a distinct stage of cancer care and developed recommendations for professionals who treat survivors, ensuring proper care to fulfill the needs of this population^5^.

Several North American health organizations have highlighted the importance of lifelong follow-up care for cancer survivors^5^. Long-term follow-up guidelines were issued by the Children Oncology Group (COG) to monitor late effects on cancer survivors in childhood^5^ ^), (^ ^6^. In its programs, the American College of Surgeons (ACOS) also emphasize the relevance of follow-up for cancer survivors, which includes a survivor care plan structured by its accredited institutions^7^.

Cancer survivors in childhood are susceptible to late effects of cancer. These health problems affect the body or mind and can occur months or even years after the end of cancer treatment^6^ ^), (^ ^8^. Late effects can include a second cancer; organ and tissue disease; growth and developmental disorders; learning and memory problems; social and psychological maladjustment; as well as changes in mood, feelings and behavior^6^ ^), (^ ^8^.

Late effects contribute to a high rate of morbidity among cancer survivors in childhood. Between 60% and 90% of these patients develop one or more chronic health conditions and may present serious complications, with risk of death during adulthood^3^ ^), (^ ^6^ ^), (^ ^9^. Such data justify the need for evaluation and long-term follow-up of persistent physical and psychosocial effects related to treatment^10^.

However, for an effective follow-up of the late effects of cancer, the transition process should be understood and monitored, considering that a disease such as cancer involves uncertainty, affecting the survivor and his/her family. Monitoring survivors in this transition requires establishing a long-term relationship between health services and adolescents in order to provide global, targeted, multidisciplinary follow-up that involves the survivor and his/her family^9^ ^), (^ ^11^. Understanding the transition can prepare health professionals for a more comprehensive approach, focused on the needs of survivors. Today, rehabilitation efforts are being increasingly used in pediatric oncology wards to support the transition of patients who return to daily routine as early as possible in order to minimize the damage caused by the treatment^10^.

In international studies, transition has been discussed as the turning point, which is the period after the end of treatment and just before becoming a survivor^12^. It is used to describe the turning point of care after the end of treatment and the return to a “normal life”^12^. In our study, the turning point refers to the transition from disease to survival, the moment when adolescents are no longer sick and become cancer survivors.

Understanding the transition from disease to survival of adolescents who had cancer is important so these aspects can be included in the planning of specific health care interventions focused on this stage of life. Therefore, this study aimed to understand the transition from disease to survival of adolescents who had experienced cancer.

## Method

### Study design

This is a secondary interpretive process that sought to answer a research question, with an objective and focus that transcends the original study^13^. We conducted this secondary analysis to understand the transition from disease to survival of adolescents who had cancer. The idea to conduct a secondary analysis emerged from questions raised in the original research, which aimed to learn more about the experience of adolescent survivors of childhood cancer. We sought to go beyond the description of how the experience is supported by the team and the family for an interpretive analysis to discover patterns of human behavior or subjective experience^14^. This analysis helped understand the strategies used by adolescents to face this transition and survive childhood cancer. This systematic approach provides resources for a deeper look at the daily routine of these adolescents who experience an extremely difficult situation and seek care strategies focused on the challenges experienced at the moment of transition, without discussing survival^14^.

Symbolic interactionism was used as the theoretical framework in this qualitative study. This theoretical framework was selected due to the possibility of understanding the “turning point” phenomenon, as it aims to understand the cause of human action and how individuals act in relation to their definitions and beliefs^15^. The meaning of the “turning point” event is attributed by individuals through relationships made during social interactions^15^.

### Study setting

Data collection in the primary study was performed from March 2018 to January 2019 using individual interviews. This study included adolescent cancer survivors who were discharged from the oncology service of a public hospital specializing in pediatric oncology in São Paulo who remain under follow-up at the outpatient clinic, and who had finished the treatment at least five years before, with no sign of recurrent disease. The period of five years has been highlighted in the literature as a sign of healing and survival^2^ ^), (^ ^3^.

The outpatient clinic selected as the study setting is a reference service in childhood cancer care. Adolescents treated at this hospital, after five years of their discharge from the oncology service with no sign of the disease are referred to this outpatient clinic, where they are monitored by a multidisciplinary team.

Participants were recruited by convenience sampling using the daily appointment schedule of the outpatient clinic, where the main researcher approached the adolescents while they were waiting for the appointment in the waiting room.

### Participants

**Figure 1:**
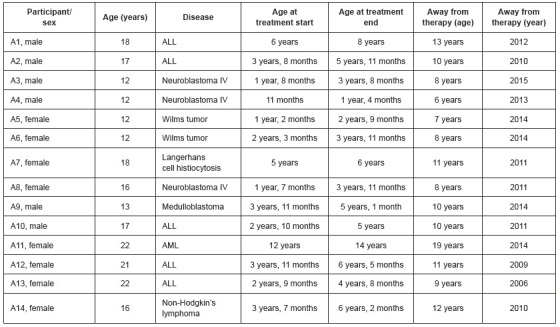
Characterization of all participants.

Fourteen adolescents aged 12 to 22 years participated in the study. Of all adolescents contacted by the researcher, two refused to participate in the study. Their diagnoses were: acute lymphoblastic leukemia (ALL), acute myeloid leukemia (AML), neuroblastoma, medulloblastoma, Langerhans cell histiocytosis, non-Hodgkin’s lymphoma, and Wilms tumor. Figure 1 shows the characterization of all participants.

São Paulo, SP, Brazil, 2019

The inclusion criteria were: individuals aged 12 to 25 years old, cancer survivors, treated at the outpatient clinic after cancer therapy, living in the city of São Paulo or in the metropolitan region, and who had no sign of the disease for at least five years. The age group of 12 to 25 years was selected considering the change in the population profile in recent years^16^, showing deferral of autonomy and intellectual maturity among young people. Although the World Health Organization (WHO) considers adolescence the period of 10 to 19 years^17^, authors have used the expanded age group from 12 to 25 years^16^.

The exclusion criteria were: adolescents with cognitive deficit, communication difficulty, hearing impairment or mutism, and adolescents with other associated diseases. This study had no participant exclusion.

### Data collection

The interviews were performed by the main researcher, who used the Original Romance technique, an intensive in-depth interview method where an interviewee narrates his/her own story, which can be considered as novelistic construction, a historical reconstitution told in first person^18^.

The guiding question for this study was: Tell me your life story as if it were a book. For younger adolescents, the researcher had to provide guidance that did not influence the narrative construction process, such as: “Tell your life story as you would like it to be presented” and “Tell me about the things that happened in your life”. The interviews lasted around 50 minutes.

Of all interviews performed, six were conducted in the waiting room of the outpatient clinic; six adolescents chose to be interviewed at home, one at the university where he studies, and one adolescent chose a shopping mall. During the interviews, we sought to ensure privacy to the adolescents.

Participants could choose to have a companion during the interview or not. Seven adolescents chose to have a companion: eight were accompanied by their mothers, one by both father and mother, one participant had an aunt, one adolescent had the husband, and one adolescent had a friend. Six participants performed the interview alone. The interviews were recorded and were later fully transcribed. Notes were made immediately after the interviews to avoid data loss and alteration.

Participants were identified by the capital letter A (adolescent) followed by the sequential number of the interview and the participant’s sex.

### Data analysis

Inductive thematic analysis was used because it is a flexible technique that allows rich and detailed data analysis. The thematic analysis had six stages^19^:

(1) Familiarization with data, which consisted of data transcription, and interview reading and rereading; (2) initial coding, when the first codes were created and grouped in a systematic manner, comparing the relevant data of every code; (3) search for patterns of related responses, grouping codes under potential themes and gathering relevant data under every potential theme; (4) review of themes, with the analysis of existing relationships between themes, data, and initial coding, generating a thematic analysis map; (5) definition and naming of themes, which comprised the data organization according to predefined themes, leading to description of the results; and (6) description of the results and the main conclusions. The analytical stage was performed by the main author and presented for discussion with the research group.

After the analysis, four themes were identified: going back to school, being able to live like other adolescents, living in the present moment, and seeking a purpose in life.

### Ethical aspects

This study was approved by the ethics committee (approval number: 68985317.3.0000.5392). An informed consent form was signed by the legal guardian of the adolescent and the researcher. The assent form was signed by the adolescent and the researcher.

### Rigor

The rigorous analysis of data produced by the researchers and supported by the narratives of interviewees ensured credibility to the study^20^ ^), (^ ^21^, the characterization of participants indicated transferability, the criteria in the checklist Consolidated Criteria for Reporting Qualitative Research (COREQ)^22^ ensured reliability, and the study limitations and positive aspects showed reflexivity of the researchers and study confirmability^20^ ^), (^ ^21^.

## Results

The end of treatment led the adolescents to a transition in life, when they were no longer cancer patients and became survivors, or individuals included in a social environment beyond the disease context. This new stage required adaptation of adolescents to the new phase of life.

Changes after the end of treatment start with a difficult adaptation to school, but, over time, adolescents get used to the new reality and seek normality in their lives. They see themselves as regular adolescents and want to live in the present moment and enjoy life here and now, seeking a purpose in life.

### Going back to school

Returning to everyday activities can be a challenging task for adolescent cancer survivors. Attending school with the effects of the treatment, such as alopecia, asthenia, and other consequences, generates fear and insecurity.

Some participants did not attend school during the treatment period. Fearing discrimination due to side effects, some parents chose to have their children out of school during cancer treatment.

*I was in kindergarten. I was in kindergarten and she* [the mother] *didn’t let me go to school without my hair. I didn’t go. So I lost one year.* (A12, female)

*I was behind the other students at school, but I attended one day or another, and in those days, my hair was falling out, there was this rejection at school. Many friends talked about it, made jokes, laughed. But it’s a phase we go through, right?* (A5, female)

*After a long period away from school, the return after the end of treatment was full of uncertainties and insecurities. Different physical aspect due to the treatment caused difficult acceptance by other children, affecting the school relationships.*

*Ah, in the beginning, it was a little difficult, because our hair falls out, and then it’s difficult for you to go to school. There’s the acceptance of people, of you being with them, with an appearance like that, different from normal life, different from what the human being is used to, right?* (A5, female)

*I used to go to school wearing a cap, because my hair had fallen out.* (A1, male)

Despite the pain and distress, the hospital was a familiar environment for them, while the school became an unpleasant place, where they were bullied due to their physical condition resulting from the treatment.

*At school it’s like any child, but I have some bullying* [...] *they call me fat. I feel sad, but I ignore it and it goes away.* (A3, male)

*At school, the children bother me, they make fun of me, they call me ‘big head’. I feel bad. I feel sad and I walk away from them. But then, when I’m here* [at the outpatient clinic]*, nobody can bother me.* (A14, male)

The transition from the hospital to a new life as survivors was challenging for the adolescents. In their narratives, they described the security they felt in the hospital environment and the reception of the health team and revealed difficulties in interacting with new people they did not know and were not touched by their life story. The transition from the hospital environment to school life caused loneliness and helplessness.

*They give us a lot of support* [the hospital team]*, when we are feeling down, discouraged. They come and tell us positive words, they encourage and talk about experiences of other people they have treated and who have recovered. Did you understand?* […] *There is always someone there by your side, like doctors, nurses, to support us, to cheer us up. But out of the hospital it’s not like that.* (A11, female)

*So, I loved the nurses, doctors, mother, family, and then I went out to a world I didn’t know, a world of strange people, people who didn’t understand my distress, they didn’t understand it because it didn’t go away. That was the most difficult part. People look, perhaps with compassion, that didn’t happen! For me, it was the hardest part.* […] *Inside the hospital for me it was like heaven and out of it, it was like a place of monsters. I had no peace, you understand? I tried, but all the time some people wanted to put me down, make me sad.* (A13, female)

Being able to live like other adolescents

Over time, the reality changed and the adolescents got used to their new life as survivors. Adaptations were made to create a normal routine for them.

Being able to live like other adolescents indicates survivors believe they can live like other adolescents who did not have cancer, being able to perform sports activities, working, and studying like any other adolescents.

*Ah, I think today my life is normal. I’m a normal girl, just like the others. Today I am just like the others. I don’t see any difference. I’m an ordinary person, I can do everything the other girls do. They are smart and so am I. Having cancer for me hasn’t changed me at all. Did you understand? It seems that I didn’t have anything. If I don’t tell about it, nobody knows […].* (A6, female)

*My life is super normal, what we went through doesn’t get in the way, I remember very little.* (A8, female)

*The desire to have an ordinary life, like any other adolescent, is a common aspect among cancer survivors. They want to leave the cancer experience behind so that it does not influence their daily activities.*

*My mother said I was healed. There was even a small party, everyone at home […] then, I could go on with my normal life.* (A9, male)

*I wanted to live as other people do, without any disease, without any health concern that something can happen. Today I live like any other person, I study, I work [pause], I play sports, I play, I run, I jump. I do everything other people can do. The disease didn’t affect anything.* (A10, male)

### Living in the present moment

The desire to leave cancer in the past and live life without thinking the disease could deprive them of something or make them different from their friends or families are attributes of study adolescents in their transition process. Survivors try to live life as best they can, focused on enjoying and valuing every day.

Living life here and now is their strategy to continue their trajectory, without allowing cancer to define them or influence their lives, living one day at a time, without the constant presence of the past.

[…] *I think that if we think so much about the future or about the past, I think there’s no need... you know, you have to live here and now, because you don’t know if you’ll be here tomorrow, so I live the present moment.* (A7, female)

*I don’t think about anything. I just live my life.* […] *I just go with the flow. I even forget that I had it. Because it doesn’t affect my daily life, except when I see the doctor... And if I get sick again, if something happens, I don’t want to know, because* [pause]*, maybe, I won’t be cured. So why would I want to know?* (A12, female)

The participants showed a different perspective of life after experiencing cancer. They showed a strong will to live and live well, without worrying about other people’s approval or the uncertainty of the future.

*I think I’m a contrarian teenager, who would rather hang out with friends, play a sport, which is a healthy thing, instead of going to a party and drinking, like kids do today.* (A2, male)

*I think that any disease, like cancer, teaches a lot* [...]*. We end up with a different essence. All children who are there, they are different, because all of us have been through something serious related to illness. But we have something in common, we are equal in terms of how we’re going to live life from now on.* (A13, female)

### Seeking a purpose in life

Searching for a meaning in life is a very important aspect in the lives of adolescent cancer survivors. They feel grateful to God and attribute the healing to a divine intervention. Adolescents believe they had a second chance and look for a purpose to be alive.

*I think that, if I went through this, it’s because at some point God knew that I would get out of this, I would take my learning to someone and I would grow very well, going through everything. It’s the second chance God has given me.* […] *I always understood that if all these things happened, it has some purpose. I didn’t survive for nothing. I’m grateful for that!* (A13, female)

*My mother says that many people have cancer and that it was a miracle from God that I got rid of this disease.* (A3, male)

*You have to be strong, right? You can’t give up, never give up, pursue what you want, because God has a purpose for everyone… you have to think positively, right? For me, I am a winner!* (A6, female)

The search for a purpose in life is seen in the intention to help sick people, as mentioned by the adolescents, who believe they can replicate the care received. They show a tendency to take health courses or volunteer services, always aiming to help others.

*I think about being able to help people, children. Like the doctors who healed me, I want to be like them too, who treat cancer* [...] *Just like the doctors could help me, I would like to be able to help someone and not let a person die.* (A10, male)

*If I manage to gather them* [the family]*, because I think it’s my mission, it would be awesome, I could help everyone*. (A4, male)

*I think I’m different in my way of being with people, of treating people well, of always saying things to their faces, of being honest, you have to be honest with people.* (A2, male)

## Discussion

A disease like cancer causes several changes in the lives of those who have this experience. When the treatment is over, the patient returns to daily activities such as attending school and other social activities. Going back to everyday life may not be an easy task for cancer survivors, since isolation and loneliness are common in this period^10^ ^), (^ ^23^.

Adolescent cancer survivors have many personal challenges after the treatment: they feel emotionally unstable, socially disconnected, and physically tired. In addition, they feel the lack of empathy from their peers^12^, which was also mentioned by the participants of this study.

The phase of social isolation, especially from school, can create significant delays in life goals of these adolescent cancer survivors. Physical and social effects of cancer treatment that appear in this stage can significantly impact well-being^24^. Inappropriate approaches or attitudes from other people, both in the school and family environment, can affect their perspectives, reducing quality of life and causing distress^23^ ^), (^ ^24^ ^), (^ ^25^ ^), (^ ^26^, changing the return to school into an obstacle.

Interaction with other adolescents is among the challenges found in returning to school activities. Support from colleagues and teachers is very important in this process of school reinsertion; however, changes in appearance cause demotivation, anxiety, and negative thoughts^26^, leading to social withdrawal^25^.

Being able to return to school is an important component of the survival period; it is associated with improved quality of life and represents the return to normal life. Adolescents and young adults who are cancer survivors^27^ significantly value living “like everyone else”, seeking a focus on their well-being and distance from future concerns^26^ ^), (^ ^27^ ^), (^ ^28^ ^), (^ ^29^. Their perceptions of a normal life are based on good social interactions, building good relationships, playing sports, going out with friends, going to school. These activities are considered by adolescents as part of their positive and healthy growth.

Living with the feeling of normality, of something culturally acceptable by society, related to feeling whole and able to set goals and have a purpose in life - this is part of a balanced life. For cancer survivors, preparation is necessary to achieve such balance.

Over time, most survivors of our study learned that, as they aged or started a new phase of their lives, they continually faced situations that brought back their cancer experience again^28^.

The possibility of new symptoms and the uncertainty regarding the future are stressors that can trigger negative emotions^29^. Feelings of uncertainty during the period of transition to survival involve multiple factors - not only medical, but social, and personal factors. They may not be limited to the disease itself, fear of recurrence and late effects, but also involve psychosocial aspects about what to expect when returning to life after treatment^12^ ^), (^ ^28^.

Continuous monitoring programs are necessary to identify possible late effects, offer emotional support and health education, and improve the quality of life of these adolescent cancer survivors^12^ ^), (^ ^29^ ^), (^ ^30^ ^), (^ ^31^ ^), (^ ^32^ ^), (^ ^33^ ^), (^ ^34^. Care management models for cancer survivors are being developed around the world to support these patients handle the consequences of the treatment and help with required adaptations in the period of transition from disease to cure^12^ ^), (^ ^28^ ^), (^ ^29^ ^), (^ ^30^ ^), (^ ^31^ ^), (^ ^32^ ^), (^ ^33^ ^), (^ ^34^.

Health professionals and patients have to be prepared for the transition from being a cancer patient to becoming a survivor^12^ ^), (^ ^24^. Maintaining the psychosocial health and psychological well-being of survivors is a task to be understood and developed by health professionals. Nurses are key professionals to support survivors in the transition period and understand the main demands and anxieties of survivors in this stage^11^.

With the end of treatment, care management is no longer focused on the disease, it is centered on well-being. Cancer survival care mainly manages comorbidities, preventing cancer recurrence and secondary cancer, and promoting quality of life^12^. Guidance and education of survivors and their families during the transition period are based on providing access to resources, support for a change in health behaviors, always aiming to achieve a state of well-being^12^.

The transition period addressed in international studies as a “turning point” can be described as the moment to return to a normal life, a restart with usual care from a primary care provider or change from disease-centered care to care focused on well-being or recovery, a return to normal health^12^.

Understanding the turning point can be used to recognize evolving portraits of the self and identify changes in the identity of adolescents, especially among young survivors, who may identify with other identities as part of the process of starting adulthood^31^.

The transition to post-treatment care can be more difficult than expected, as some adolescents try to process the cancer experience and find a “new identity”^33^. Factors such as physical and psychosocial challenges associated with continuous symptoms related to cancer or treatment lead adolescent survivors to seek self-reconstruction^33^. Survivors may seek to change their identities due to the need for psychological well-being and improved self-esteem^31^.

Childhood cancer is an experience that is closely associated with a process of identity construction or reconstruction^28^ ^), (^ ^29^. The past identity may reflect a struggle, an identity paradox between not wanting cancer to define the survivor’s current life and creating a present identity, which includes reflecting on and honoring the cancer experience while seeking to be distant from that experience, taking the survivor to post-cancer life^29^.

Childhood cancer survivors have their identities altered during the course of the disease. The adolescents of this study, despite believing that they are not represented by the disease, knew that cancer was very important in their lives^31^. They could see the biographical disruptions of cancer have changed over time, moving from a need for self-protection and not being destroyed by the disease to defining their cancer disease as partial integration into their sense of self.

After the end of treatment, as patients restart life, self-identity is reformed, reintegrated, and renegotiated, sometimes as a survivor, victim, or just a person who has had cancer. This moment of identity reform involves recovering the ability to see oneself beyond cancer^12^.

Survivors acquired a more flexible and optimistic way of thinking. These behaviors are attributed to the cancer experience and described in the literature as traits of personal growth and maturity^24^.

Childhood cancer survivors show a high level of satisfaction with their current lives^28^ ^), (^ ^35^, and this satisfaction may be related to the way they identify themselves as adults^24^. Promoting the ability to achieve a sense of adulthood can be impactful among adolescent cancer survivors who struggle to find normality in their lives. Consequently, it can help them handle the consequences of the cancer experience^24^.

Gratitude to God is also important in the statements of adolescents, who reveal having had a second chance, a divine gift or a blessing. Adolescents believe they were healed for a divine purpose and show a high perception of hope in life^36^. Similar accounts have been described in a study conducted with the indigenous population in Canada, in which survivors attributed cure to a gift, a plan from the Creator or considered it part of a spiritual journey^37^.

Perception of hope among childhood cancer survivors was correlated with psychological well-being and quality of life in a study conducted in Hong Kong. This study found that lower levels of hope are associated with depressive symptoms and low self-esteem^33^. Belief in the divine destiny and the search for a purpose become factors that encourage adolescents, minimizing the negative effects related to survival.

In the general population, spiritual well-being is related to the presence of a purpose or meaning in life and has been associated with reduced anxiety and other health benefits, such as improved memory, lower risk of disability, and lower mortality rate^38^. Then, a stronger sense of meaning in cancer survivors is also associated with better quality of life, better physical and emotional well-being, and mental balance^38^.

The literature shows that some people find more meaning in their lives after the cancer experience^31^ ^), (^ ^36^ ^), (^ ^37^ ^), (^ ^38^ ^), (^ ^39^. An existential meaning is sought, integrating the changes and creating a new self in the process of becoming a cancer survivor. This search for meaning in life or process of creating meaning is linked with psychological well-being. Patients may develop the so-called “existential distress in cancer” as the “search for meaning” after experiencing a disease such as cancer^36^ ^), (^ ^37^ ^), (^ ^38^ ^), (^ ^39^. However, this process is only beneficial when meaning is found, while a search without finding a meaning can negatively affect the well-being of the survivor^39^.

The search for meaning can reach segments of altruism. Adolescents tend to volunteer and help other sick people; they also reveal the desire to take health courses aiming to help cancer patients. Young people who experience cancer tend to choose university health courses encouraged by their experiences^28^. Some survivors support causes such as brain tumors and lung cancer, and others engaged in the struggle for policies and care practices to improve the visibility and understanding of cancer patients and survivors^31^.

Survivors seek to live the present moment and enjoy what life can offer them. Adolescents in our study showed stronger appreciation of life; cancer has encouraged spirituality and existential aspects of human functioning. It is probably due to a direct association of cancer with death, resulting in a feeling of proximity to finitude and the belief of a new opportunity for life.

Seeing life as a second chance, adolescents try to live without thinking about the future, living the present moment, without worrying about events that may be out of their control. It becomes an alternative to deal with the uncertainties of life with less distress^28^.

Adolescent cancer survivors acquire new priorities in life, new values, and new philosophies after the disease. The cancer experience allowed them to understand the priceless value of life^28^; the experience of childhood cancer made them understand the importance of new opportunities and experience the feeling of gratitude, encouraging them to seek a more meaningful life with clear goals^36^.

This study helps expand the knowledge about adolescents who experience the transition from the end of treatment to healing, a topic that is still little explored in the Brazilian context. It is important to expand programs for survivors, which must be articulated with hospital services and community actions in order to improve the well-being and quality of life of this population.

As a study limitation, data collection was conducted in only one cancer treatment service in the city of São Paulo. Further studies should address the experience of adolescents and young people who have undergone cancer treatment in different health centers and in different cities.

## Conclusion

This study helped understand the transition experienced by adolescent cancer survivors and the several challenges faced in the transition from cancer patients to survivors. Life after cancer proved to be full of insecurities, difficulties, and challenges.

This study allowed researchers to understand more about this universe of experience after the cure of such a significant disease and showed a number of tools and strategies adopted by these adolescents to strengthen and recognize themselves as individuals.

After the disease, adolescents acquired new priorities and new values in life. The cancer experience also anticipated maturity, developing a feeling of gratitude to God and their families. For them, there is a purpose for their survival, which makes them more altruistic, seeking to live well because they believe they have had a second chance.

The development of health actions and programs to monitor these adolescent cancer survivors is essential due to an increase in the number of survivors worldwide. Incentives must be granted to research, practical proposals, and public policies to assist this population. Studies are recommended with children and adolescents who are cancer survivors in order to identify health needs and difficulties of this population and propose and test new survivor monitoring methods, through the expansion of care ranging from self-management to tracking late effects. Also, models should be proposed for shared care between the health service of origin of the adolescent and the primary health care service so that survivor monitoring is fully and effectively performed.

Our study may contribute as a guide for further investigation with adolescent cancer survivors. The health demands of this population require a better understanding for the development of specific and individualized care.

This study highlighted the importance of keeping the relationship between the health team and cancer survivors, even after the end of treatment, as this relationship of trust with the health team that participated in their treatment encourage adolescents to continue the outpatient follow-up. This fact reinforces the importance of reciprocity and partnership in care and transparency in relationships, especially when providing care to children and adolescents.

## References

[1] Turcotte LM, Neglia JP, Reulen RC, Ronckers CM, Leewen FE, Morton LM (2018). Risk, Risk Factors, and Surveillance of Subsequent Malignant Neoplasms in Survivors of Childhood Cancer: A Review. J Clin Oncol.

[2] Howlader N, Noone AM, Krapcho M, Miller D, Bispo K, Kosary CL (2020). SEER Cancer Statistics Review, 1975-2017.

[3] Miller KD, Nogueira L, Devasia T, Mariotto AB, Yabroff KR, Jemal A (2022). Cancer treatment and survivorship statistics, 2022. CA Cancer J Clin.

[4] Mullan F. (1985). Seasons of survival: reflections of a physician with cancer. N Engl J Med.

[5] National Cancer Institute (2020). About Cancer Survivorship Research: Survivorship Definitions.

[6] National Cancer Institute (2022). Efeitos tardios do tratamento para câncer infantil (PDQ®).

[7] American College of Surgeons (2020). Commission on cancer.

[8] Erdmann F, Frederiksen LE, Bonaventure A, Mader L, Hasle H, Robison LL (2021). Childhood cancer: survival, treatment modalities, late effects and improvements over time. Cancer Epid.

[9] Norsker FN, Rechnitzer C, Cederkvist L, Holmqvist AS, Tryggvadottir L, Madanat-Harjuoja LM (2018). Somatic late effects in 5-year survivors of neuroblastoma: a population-based cohort study within the Adult Life after Childhood Cancer in Scandinavia study. Int J Cancer.

[10] Andrés-Jensen L, Larsen HB, Johansen C, Frandsen TL, Schmiegelow K, Wahlberg A (2020). Everyday life challenges among adolescent and young adult survivors of childhood acute lymphoblastic leukemia: An indepth qualitative study. Psycho-Oncol.

[11] Neris RR, Nascimento LC (2021). Childhood cancer survival: emerging reflections on pediatric oncology nursing. Rev Esc Enferm USP.

[12] Wood SK (2018). Transition to cancer survivorship: a concept analysis. ANS Adv Nurs Sci.

[13] Heaton J (2008). Secondary analysis of qualitative data: An overview. Historical Social Research/Historische Sozialforschung.

[14] Thorne S (2008). Interpretive description: Developing qualitative inquiry.

[15] Charon JM (2010). Symbolic interacionism: an introduction, an interpretation, an integration.

[16] Cousijn J, Luijten M, Ewing SWF (2018). Adolescent resilience to addiction: a social plasticity hypothesis. Lancet Child Adolesc Health.

[17] Word Health Organization (2021). Adolescent and young adult health.

[18] Mazorra L (2009). A construção de significados atribuídos à morte de um ente querido e o processo de luto.

[19] Braun V, Clarke V (2019). Reflecting on reflexive thematic analysis. Qual Res Sport Exerc Health.

[20] Doyle L, McCabe C, Keogh B, Brady A, McCann M (2020). An overview of the qualitative descriptive design within nursing research. J Res Nurs.

[21] Dodgson JE (2019). Reflexivity in Qualitative Research. J Hum Lact.

[22] Tong A, Sainsbury P, Craig J (2007). Consolidated criteria for reporting qualitative research (COREQ): a 32-item checklist for interviews and focus groups. Int J Qual Health Care.

[23] Lea S, Martins A, Fern LA, Bassett M, Cable M, Doig G (2020). The support and information needs of adolescents and young adults with cancer when active treatment ends. BMC Cancer.

[24] Kim Y, Ritt-Olson A, Tobin J, Haydon M, Milan J (2022). Beyond depression: correlates of well-being in young adult survivors of childhood cancers. J Cancer Surviv.

[25] Braga TR, Mattos CX, Cabral IE (2021). Participatory health education on school (re)inclusion of the adolescent cancer survivor. Rev Bras Enferm.

[26] Yamasaki F (2022). Adolescent and young adult brain tumors: current topics and review. Int J Clin Oncol.

[27] Psihogios AM, Schwartz LA, Deatrick JA, Hoeve ESV, Anderson LM, Wartmsn EC (2019). Preferences for cancer survivorship care among adolescents and young adults who experienced healthcare transitions and their parents. J Cancer Surviv.

[28] Belpame N, Kars MC, Deslypere E, Rober P, Hecke AV, Verhaeghe S (2019). Living as a Cancer Survivor: A Qualitative Study on the Experiences of Belgian Adolescents and Young Adults (AYAs) after Childhood Cancer. J Ped Nurs.

[29] Darabos K, Ford JS (2020). “Basically, You Had Cancer and Now You Don’t”: Exploring the Meaning of Being a “Cancer Survivor” Among Adolescents and Young Adult Cancer Survivors. J Adolesc Young Adult Oncol.

[30] Denzler S, Otth M, Scheinemann K (2020). Aftercare of Childhood Cancer Survivors in Switzerland: Protocol for a Prospective Multicenter Observational Study. JMIR Res Protoc.

[31] Hammond C, Teucher U (2017). An abundance of selves: young adults’ narrative identities while living with cancer. Cancer Nurs.

[32] Belpame N, Kars MC, Deslypere E, Rober P, VanHecke A, Verhaeghe S (2021). Coping Strategies of Adolescent and Young Adult Survivors of Childhood Cancer: A Qualitative Study. Cancer Nurs.

[33] Jin Z, Griffith MA, Rosenthal AC. (2021). Identifying and Meeting the Needs of Adolescents and Young Adults with Cancer. Curr Oncol Rep.

[34] Ho L, Li WHC, Cheng AT, Ho E, Lam K, Chiu SY (2021). Relationships among hope, psychological well-being and health-related quality of life in childhood cancer survivors. J Health Psychol.

[35] Datillo TM, Olshefsky RS, Nahata L, Hansen-Moore JA, Gerhardt CA, Lehmann V (2021). Growing up after childhood cancer: maturity and life satisfaction in young adulthood. Sup Care Cancer.

[36] Nilsen M, Stalsberg R, Sand K, Haugan G, Reidunsdatter RJ (2021). Meaning Making for Psychological Adjustment and Quality of Life in Older Long-Term Breast Cancer Survivors. Front Psychol.

[37] Gifford W, Thomas O, Thomas R, Grandpierre V, Ukagwu C (2019). Spirituality in cancer survivorship with First Nations people in Canada. Sup Care Cancer.

[38] Sleight AG, Boyd P, Klein WMP, Jensen RE (2021). Spiritual peace and life meaning may buffer the effect of anxiety on physical well-being in newly diagnosed cancer survivors. Psychooncology.

[39] Krok D, Telka E (2018). Meaning in life in cancer patients: relationships with illness perception and global meaning changes. Health Psyc Report.

